# Supplementary material for: Preconception syphilis seroprevalence and association with duration of marriage and age among married individuals in Guangdong Province, China: A population-based cross-sectional study
Source: PLoS Negl Trop Dis. 2022 Nov 28;16(11):e0010884. doi: 10.1371/journal.pntd.0010884 (PMC9731487; doi:10.1371/journal.pntd.0010884)
Supplement: S1 STROBE Checklist — (DOCX) [file pntd.0010884.s001.docx]

**S1 STROBE Checklist.**

|  | Item No | Recommendation | Section, paragraph Response |
| --- | --- | --- | --- |
| **Title and abstract** | 1 | (*a*) Indicate the study’s design with a commonly used term in the title or the abstract | Title |
|  |  | (*b*) Provide in the abstract an informative and balanced summary of what was done and what was found | Abstract: Methods & findings. |
| Introduction | | |  |
| Background/ rationale | 2 | Explain the scientific background and rationale for the investigation being reported | Abstract: Background. Introduction. |
| Objectives | 3 | State specific objectives, including any prespecified hypotheses | Abstract: Background. Introduction: final paragraph. |
| Methods | | |  |
| Study design | 4 | Present key elements of study design early in the paper | Abstract: Methods and findings. Methods: Study design and participants |
| Setting | 5 | Describe the setting, locations, and relevant dates, including periods of recruitment, exposure, follow-up, and data collection | Methods: Study area. Methods: Study design and participants, Outcome and covariates. S1 Fig. |
| Participants | 6 | (*a*) Give the eligibility criteria, and the sources and methods of selection of participants | Methods: Study area, Study design and participants. Results: paragraph 1. S1 Fig. |
| Variables | 7 | Clearly define all outcomes, exposures, predictors, potential confounders, and effect modifiers. Give diagnostic criteria, if applicable | Methods: Measures and covariates, Statistical analysis. Table 3. S4-S6 Fig. |
| Data sources/ measurement | 8 | For each variable of interest, give sources of data and details of methods of assessment (measurement). Describe comparability of assessment methods if there is more than one group | Methods: Study design and participants, Measures and covariates. |
| Bias | 9 | Describe any efforts to address potential sources of bias | Methods: Measures and covariates, Statistical analysis. |
| Study size | 10 | Explain how the study size was arrived at | Methods: Study design and participants. Results: first paragraph. S1 Fig. |
| Quantitative variables | 11 | Explain how quantitative variables were handled in the analyses. If applicable, describe which groupings were chosen and why | Methods: Measures and covariates. |
| Statistical methods | 12 | (*a*) Describe all statistical methods, including those used to control for confounding | Methods: Statistical analysis. |
|  |  | (*b*) Describe any methods used to examine subgroups and interactions | Methods: Statistical analysis (final paragraph). S4-S6 Fig. |
|  |  | (*c*) Explain how missing data were addressed | Methods: Statistical analysis (second paragraph). |
|  |  | (*d*) If applicable, describe analytical methods taking account of sampling strategy | Not applicable. |
|  |  | (*e*) Describe any sensitivity analyses | Methods: Statistical analysis (final paragraph). S3 Fig. |
| Results | | |  |
| Participants | 13 | (a) Report numbers of individuals at each stage of study—eg numbers potentially eligible, examined for eligibility, confirmed eligible, included in the study, completing follow-up, and analysed | Results: first paragraph. S1 Fig. |
|  |  | (b) Give reasons for non-participation at each stage | S1 Fig. |
|  |  | (c) Consider use of a flow diagram | S1 Fig. |
| Descriptive data | 14* | (a) Give characteristics of study participants (eg demographic, clinical, social) and information on exposures and potential confounders | Results: first paragraph. Table 1. S1-S2 Table. |
|  |  | (b) Indicate number of participants with missing data for each variable of interest | Table 1. S1-S2 Table. |
| Outcome data | 15* | Report numbers of outcome events or summary measures | Table 1. S1-S2 Table. |
| Main results | 16 | (*a*) Give unadjusted estimates and, if applicable, confounder-adjusted estimates and their precision (eg, 95% confidence interval). Make clear which confounders were adjusted for and why they were included | Table 2. Fig 4. S3 Table and S2 Figure. |
|  |  | (*b*) Report category boundaries when continuous variables were categorized | Table 1-Table 3. |
|  |  | (*c*) If relevant, consider translating estimates of relative risk into absolute risk for a meaningful time period | Not Applicable |
| Other analyses | 17 | Report other analyses done—eg analyses of subgroups and interactions, and sensitivity analyses | Table 3. S3-S6 Fig. |
| Discussion | | |  |
| Key results | 18 | Summarise key results with reference to study objectives | Abstract: Methods and findings, Conclusions. Discussion: paragraphs 1-5. |
| Limitations | 19 | Discuss limitations of the study, taking into account sources of potential bias or imprecision. Discuss both direction and magnitude of any potential bias | Abstract: Methods and findings. Discussion: Strengths and limitations. |
| Interpretation | 20 | Give a cautious overall interpretation of results considering objectives, limitations, multiplicity of analyses, results from similar studies, and other relevant evidence | Discussion |
| Generalis-ability | 21 | Discuss the generalisability (external validity) of the study results | Discussion: Strengths and limitations. |
| Other information | | |  |
| Funding | 22 | Give the source of funding and the role of the funders for the present study and, if applicable, for the original study on which the present article is based | Provided during submission process |
