# Supplementary material for: Preconception syphilis seroprevalence and association with duration of marriage and age among married individuals in Guangdong Province, China: A population-based cross-sectional study
Source: PLoS Negl Trop Dis. 2022 Nov 28;16(11):e0010884. doi: 10.1371/journal.pntd.0010884 (PMC9731487; doi:10.1371/journal.pntd.0010884)
Supplement: S1 Table — (DOCX) [file pntd.0010884.s002.docx]

**S1 Table. Sociodemographic characteristics and seroprevalence of syphilis among married individuals aged 21-49 who participated in NFPHEP in Guangdong, 2014-2019.**

| **Characteristics** | **Number of screened (%) ^#^** | **Number of seropositivity (%) ^#^** | **Seropositive rate**  **per 100 000 (95% *CI*)** |
| --- | --- | --- | --- |
| **All participants** | 4,826,214 (100) | 12,867 (100) | 266.61 (262.03-271.24) |
| **Gender*** |  |  |  |
| Male | 2,398,914 (49.7) | 6,110 (47.5) | 254.70 (248.37-261.15) |
| Female | 2,427,300 (50.3) | 6,757 (52.5) | 278.38 (271.79-285.07) |
| **Age*** |  |  |  |
| 21-24 | 943,829 (19.6) | 2,154 (16.7) | 228.22 (218.70-238.04) |
| 25-29 | 2,186,185 (45.3) | 4,090 (31.8) | 187.08 (181.40-192.90) |
| 30-34 | 1,051,497 (21.8) | 2,754 (21.4) | 261.91 (252.25-271.85) |
| 35-39 | 425,198 (8.8) | 2,145 (16.7) | 504.47 (483.45-526.17) |
| 40-44 | 167,472 (3.5) | 1,243 (9.7) | 742.21 (701.66-784.48) |
| 45-49 | 52,033 (1.1) | 481 (3.7) | 924.41 (843.97-1010.40) |
| **Ethnicity*** |  |  |  |
| Han | 4,589,320 (95.1) | 12,193 (94.8) | 265.68 (261.00-270.43) |
| Minority | 39,728 (0.8) | 145 (1.1) | 364.98 (308.08-429.32) |
| Missing | 197,166 (4.1) | 529 (4.1) | 268.30 (245.95-292.14) |
| **Education*** |  |  |  |
| Primary school or below | 95,406 (2.0) | 513 (4.0) | 537.70 (492.28-586.17) |
| Junior high school | 1,328,985 (27.5) | 4,854 (37.7) | 365.24 (355.07-375.62) |
| Senior high school | 1,176,472 (24.4) | 3,221 (25.0) | 273.78 (264.43-283.38) |
| College or above | 1,628,103 (33.7) | 2,464 (19.1) | 151.34 (145.43-157.43) |
| Missing | 597,248 (12.4) | 1,815 (14.1) | 303.89 (290.11-318.16) |
| **Occupation*** |  |  |  |
| Farmer | 969,280 (20.1) | 3,478 (27.0) | 358.82 (347.04-370.91) |
| Workers | 1,147,034 (23.8) | 2,985 (23.2) | 260.24 (251.01-269.72) |
| Businessman | 297,340 (6.2) | 813 (6.3) | 273.42 (255.00-292.83) |
| Service personnel | 409,000 (8.5) | 1,073 (8.3) | 262.35 (246.92-278.48) |
| Civil servant | 911,844 (18.9) | 1,429 (11.1) | 156.72 (148.71-165.04) |
| Others | 338,471 (7.0) | 776 (6.0) | 229.27 (213.45-245.94) |
| Missing | 753,245 (15.6) | 2,313 (18.0) | 307.07 (294.72-319.81) |
| **Household registration*** |  |  |  |
| Rural | 3,512,075 (72.8) | 9,950 (77.3) | 283.31 (277.78-288.91) |
| Urban | 1,309,574 (27.1) | 2,912 (22.6) | 222.36 (214.38-230.57) |
| Missing | 4,565 (0.1) | 5 (0.0) | 109.53 (35.57-255.42) |
| **Migrant population*** |  |  |  |
| No | 4,032,434 (83.6) | 10,552 (82.0) | 261.68 (256.72-266.71) |
| Yes | 792,265 (16.4) | 2,310 (18.0) | 291.57 (279.83-303.67) |
| Missing | 1,515 (0.0) | 5 (0.0) | 330.03 (107.24-768.49) |
| **Cigarette smoking*** |  |  |  |
| No | 4,126,965 (85.5) | 10,316 (80.2) | 249.97 (245.18-254.82) |
| Yes | 661,804 (13.7) | 2,427 (18.9) | 366.72 (352.33-381.55) |
| Missing | 37,445 (0.8) | 124 (1.0) | 331.15 (275.51-394.7) |
| **Alcohol drinking*** |  |  |  |
| No | 3,734,558 (77.4) | 9,806 (76.2) | 262.57 (257.42-267.81) |
| Yes | 1,049,885 (21.8) | 2,938 (22.8) | 279.84 (269.84-290.12) |
| Missing | 41,771 (0.9) | 123 (1.0) | 294.46 (244.79-351.24) |
| **Ever used drugs*** |  |  |  |
| No | 4,774,080 (98.9) | 12,687 (98.6) | 265.75 (261.16-270.4) |
| Yes | 773 (0.0) | 14 (0.1) | 1811.13 (993.61-3020.11) |
| Missing | 51,361 (1.1) | 166 (1.3) | 323.2 (275.97-376.18) |
| **Syphilis seropositivity of spouses*** |  |  |  |
| Negative | 4,662,448 (96.6) | 11,065 (86.0) | 237.32 (232.93-241.77) |
| Positive | 12,297 (0.3) | 1,168 (9.1) | 9498.25 (8985.61-10030.15) |
| Missing | 151,469 (3.1) | 634 (4.9) | 418.57 (386.68-452.38) |
| **Region*** |  |  |  |
| Pearl River Delta | 2406,696 (49.9) | 6,123 (47.6) | 254.42 (248.10-260.85) |
| East Wing | 627,956 (13.0) | 1,805 (14.0) | 287.44 (274.37-300.97) |
| West Wing | 1,037,775 (21.5) | 2,721 (21.1) | 262.20 (252.46-272.21) |
| Mountainous Area | 753,787 (15.6) | 2,218 (17.2) | 294.25 (282.16-306.72) |
| **Duration of marriage (years)*** |  |  |  |
| 0 | 1,566,318 (32.5) | 3,833 (29.8) | 244.71 (237.05-252.57) |
| 0.1-1.0 | 1,112,045 (23.0) | 2,645 (20.6) | 237.85 (228.89-247.07) |
| >1.0 | 1,471,135 (30.5) | 4,523 (35.2) | 307.45 (298.58-316.51) |
| Missing | 676,716 (14.0) | 1,866 (14.5) | 275.74 (263.41-288.51) |
| **Previous pregnancy (Female)*** |  |  |  |
| No | 1,451,616 (59.8) | 3,546 (52.5) | 244.28 (236.32-252.43) |
| Yes | 962,304 (39.6) | 3,179 (47.0) | 330.35 (319.00-342.00) |
| Missing | 13,380 (0.6) | 32 (0.5) | 239.16 (163.64-337.46) |

Notes: Duration of marriage referred to the time difference (in years) between syphilis screening and marriage registration. Participants in the first subgroup (0 year) are newly married couples, and the duration of marriage was no more than 18 days. Moreover, the duration of marriage between 19 days and 365 days was considered to be “0.1-1.0 years”. Abbreviations: NFPHEP, National Free Preconception Health Examination Project; *CI*, confidence interval.

^#^ Data was presented as No. (%); reported percentages are composition ratios of each horizontal item.

* *P* < 0.05
