# Supplementary material for: Preconception syphilis seroprevalence and association with duration of marriage and age among married individuals in Guangdong Province, China: A population-based cross-sectional study
Source: PLoS Negl Trop Dis. 2022 Nov 28;16(11):e0010884. doi: 10.1371/journal.pntd.0010884 (PMC9731487; doi:10.1371/journal.pntd.0010884)
Supplement: S2 Table — (DOCX) [file pntd.0010884.s003.docx]

**S2 Table. Characteristics of married female** **aged 21-49 who participated in NFPHEP in Guangdong,** **according to age categories 2014-2019.**

| **Characteristics** | **Aged 21-24 years (N=634,667) ^#^** | **Aged 25-29years (N=1,100,736) ^#^** | **Aged 30-34 years (N=432,253) ^#^** | **Aged 35-39 years (N=179,923) ^#^** | **Aged 40-44 years (N=63,336) ^#^** | **Aged 45-49 years (N=16,385) ^#^** |
| --- | --- | --- | --- | --- | --- | --- |
| **Ethnicity*** |  |  |  |  |  |  |
| Han | 603,103(95.0) | 1,048,754(95.3) | 408,979(94.6) | 170,097(94.5) | 59,704(94.3) | 15,362(93.8) |
| Minority | 4,104(0.6) | 9,220(0.8) | 5,599(1.3) | 2,438(1.4) | 677(1.1) | 148(0.9) |
| Missing | 27,460(4.3) | 42,762(3.9) | 17,675(4.1) | 7,388(4.1) | 2,955(4.7) | 875(5.3) |
| **Education level*** |  |  |  |  |  |  |
| Primary school or below | 17,072(2.7) | 19,644(1.8) | 7,073(1.6) | 3,176(1.8) | 2,049(3.2) | 1,139(7.0) |
| Junior high school | 253,019(39.9) | 265,044(24.1) | 96,535(22.3) | 38,833(21.6) | 16,579(26.2) | 1,6,380(38.9) |
| Senior high school | 178,461(28.1) | 249,940(22.7) | 88,136(20.4) | 38,650(21.5) | 13,447(21.2) | 3,325(20.3) |
| College or above | 110,654(17.4) | 433,716(39.4) | 182,904(42.3) | 74,193(41.2) | 22,051(34.8) | 3,358(20.5) |
| Missing | 75,461(11.9) | 132,392(12.0) | 57,605(13.3) | 25,071(13.9) | 9,210(14.5) | 2,183(13.3) |
| **Occupation*** |  |  |  |  |  |  |
| Farmer | 173,029(27.3) | 213,033(19.4) | 71,725(16.6) | 27,715(15.4) | 11,851(18.7) | 4,405(26.9) |
| Workers | 165,390(26.1) | 237,142(21.5) | 89,357(20.7) | 34,673(19.3) | 11,134(17.6) | 3,102(18.9) |
| Businessman | 31,413(4.9) | 46,198(4.2) | 17,778(4.1) | 7,432(4.1) | 2,136(3.4) | 452(2.8) |
| Service personnel | 51,399(9.0) | 100,577(9.1) | 36,850(8.5) | 13,442(7.5) | 4,240(6.7) | 1,092(6.7) |
| Civil servant | 61,062(9.6) | 248,363(22.6) | 108,713(25.2) | 51,216(28.5) | 17,657(27.9) | 3,107(19.0) |
| Others | 50,157(7.9) | 86,229(7.8) | 35,593(8.2) | 14,256(7.9) | 4,948(7.8) | 1,348(8.2) |
| Missing | 96,217(15.2) | 169,194(15.4) | 72,237(16.7) | 31,189(17.3) | 11,370(18.0) | 2,879(17.6) |
| **Household registration*** |  |  |  |  |  |  |
| Rural | 562,418(88.6) | 822,849(74.8) | 277,879(64.3) | 91,981(51.1) | 28,650(45.2) | 8,123(49.6) |
| Urban | 72,009(11.3) | 277,207(25.2) | 153,791(35.6) | 87,519(48.6) | 34,569(54.6) | 8,250(50.4) |
| Missing | 240(0.0) | 680(0.1) | 583(0.1) | 423(0.2) | 117(0.2) | 12(0.1) |
| **Cigarette smoking*** |  |  |  |  |  |  |
| No | 627,976(98.9) | 1,087,613(98.8) | 426,085(98.6) | 177,056(98.4) | 62,314(98.4) | 16,140(98.5) |
| Yes | 1,852(0.3) | 3,667(0.3) | 2,093(0.5) | 1,043(0.6) | 380(0.6) | 110(0.7) |
| Missing | 4,839(0.8) | 9,456(0.9) | 4,075(0.9) | 1,824(1.0) | 642(1.0) | 135(0.8) |
| **Alcohol drinking*** |  |  |  |  |  |  |
| No | 592,544(93.4) | 994,996(90.4) | 385,907(89.3) | 160,353(89.1) | 56,768(89.6) | 14,921(91.1) |
| Yes | 36,053(5.7) | 94,683(8.6) | 41,796(9.7) | 17,574(9.8) | 5,890(9.3) | 1,311(8.0) |
| Missing | 6,070(1.0) | 11,057(1.0) | 4,550(1.1) | 1,996(1.1) | 678(1.1) | 153(0.9) |
| **Ever used drugs*** |  |  |  |  |  |  |
| No | 627,880(98.9) | 1,088,198(98.9) | 426,839(98.7) | 177,495(98.7) | 62,484(98.7) | 16,196(98.8) |
| Yes | 44(0.0) | 66(0.0) | 46(0.0) | 17(0.0) | 12(0.0) | 1(0.0) |
| Missing | 6,743(1.1) | 12,472(1.1) | 5,368(1.2) | 2,411(1.3) | 840(1.3) | 188(1.1) |
| **Syphilis seropositivity of spouses*** |  |  |  |  |  |  |
| Negative | 615,939(97.0) | 1,059,468(96.3) | 405,725(93.9) | 164,223(91.3) | 53,912(85.1) | 11,572(70.6) |
| Positive | 1,252(0.2) | 1,896(0.2) | 1,293(0.3) | 964(0.5) | 458(0.7) | 93(0.6) |
| Missing | 17,476(2.8) | 39,372(3.6) | 25,235(5.8) | 14,736(8.2) | 8,966(14.2) | 4,720(28.8) |
| **Migrant population*** |  |  |  |  |  |  |
| No | 539,023(84.9) | 882,934(80.2) | 341,988(79.1) | 151,949(84.5) | 57,011(90.0) | 15,197(92.7) |
| Yes | 95,512(15.0) | 217,563(19.8) | 90,145(20.9) | 27,921(15.5) | 6,305(10.0) | 1,184(7.2) |
| Missing | 132(0.0) | 239(0.0) | 120(0.0) | 53(0.0) | 20(0.0) | 4(0.0) |
| **Region*** |  |  |  |  |  |  |
| Pearl River Delta | 212,882(33.5) | 544,881(49.5) | 278,876(64.5) | 126,622(70.4) | 43,441(68.6) | 11,845(72.3) |
| East Wing | 128,086(20.2) | 142,683(13.0) | 29,001(6.7) | 7,615(4.2) | 1,817(2.9) | 418(2.6) |
| West Wing | 174,440(27.5) | 247,448(22.5) | 70,648(16.3) | 19,852(11.0) | 5,841(9.2) | 747(4.6) |
| Mountainous Area | 119,259(18.8) | 165,724(15.1) | 53,728(12.4) | 25,834(14.4) | 12,237(19.3) | 3,375(20.6) |
| **Previous pregnancy*** |  |  |  |  |  |  |
| No | 484,287(76.3) | 769,317(69.9) | 159,916(37.0) | 29,555(16.4) | 7,029(11.1) | 1,512(9.2) |
| Yes | 146,800(23.1) | 324,885(29.5) | 270,261(62.5) | 149,544(83.1) | 56,021(88.5) | 14,793(90.3) |
| Missing | 3,580(0.6) | 6,534(0.6) | 2,076(0.5) | 824(0.5) | 286(0.5) | 80(0.5) |
| **Duration of marriage (years)*** |  |  |  |  |  |  |
| 0 | 298,983(47.1) | 372,720(33.9) | 73,687(17.0) | 17,173(9.5) | 4,409(7.0) | 733(4.5) |
| 0.1-1.0 | 179,250(28.2) | 296,852(27.0) | 62,496(14.5) | 14,145(7.9) | 3,485(5.5) | 614(3.7) |
| >1.0 | 61,362(9.7) | 277,985(25.3) | 242,119(56.0) | 124,466(69.2) | 45,175(71.3) | 11,766(71.8) |
| Missing | 95,072(15.0) | 153,179(13.9) | 53,951(12.5) | 24,139(13.4) | 10,267(16.2) | 3,272(20.0) |

Notes: Duration of marriage referred to the time difference (in years) between syphilis screening and marriage registration. Participants in the first subgroup (0 year) are newly married couples, and the duration of marriage was no more than 18 days. Moreover, the duration of marriage between 19 days and 365 days was considered to be “0.1-1.0 years”.

Abbreviations: NFPHEP, National Free Preconception Health Examination Project; N, number.

^#^ Data was presented as No. (%); reported percentages are composition ratios of each horizontal item.

* *P* < 0.001
