# Supplementary material for: Preconception syphilis seroprevalence and association with duration of marriage and age among married individuals in Guangdong Province, China: A population-based cross-sectional study
Source: PLoS Negl Trop Dis. 2022 Nov 28;16(11):e0010884. doi: 10.1371/journal.pntd.0010884 (PMC9731487; doi:10.1371/journal.pntd.0010884)
Supplement: S4 Table — (DOCX) [file pntd.0010884.s005.docx]

**S4 Table**. **Preconception syphilis seroprevalence among married individuals aged 21-49 years by calendar year.**

|  | **All participants** | | | **Married male** | | | **Married female** | | |
| --- | --- | --- | --- | --- | --- | --- | --- | --- | --- |
| **Calendar year** | **Number of screened** | **Number of seropositivity** | **Seropositive rate per 100 000 (95% *CI*)** | **Number of screened** | **Number of seropositivity** | **Seropositive rate per 100 000 (95% *CI*)** | **Number of screened** | **Number of seropositivity** | **Seropositive rate per 100 000 (95% *CI*)** |
| 2014 | 870,316 | 2546 | 292.54 (281.31-304.09) | 433,196 | 1266 | 292.25 (276.41-308.75) | 437,120 | 1280 | 293.69 (277.86-310.18) |
| 2015 | 785,899 | 2057 | 261.74 (250.58-273.27) | 393,743 | 1020 | 259.05 (243.43-275.41) | 392,156 | 1037 | 264.44 (248.62-280.99) |
| 2016 | 983,995 | 2844 | 289.03 (278.53-299.82) | 487,841 | 1468 | 300.92 (285.76-316.66) | 496,154 | 1376 | 277.33 (262.91-292.34) |
| 2017 | 865,420 | 2212 | 255.60 (245.08-266.45) | 427,852 | 994 | 232.32 (218.13-247.19) | 437,568 | 1218 | 278.36 (262.98-294.39) |
| 2018 | 760,492 | 1863 | 244.97 (234.00-256.33) | 377,541 | 793 | 210.04 (195.70-225.15) | 382,951 | 1070 | 279.41 (262.96-296.61) |
| 2019 | 560,092 | 1345 | 240.14 (227.50-253.29) | 278,741 | 569 | 204.13 (187.73-221.58) | 281,351 | 776 | 275.81 (256.79-295.86) |

Abbreviations: *CI*, confidence interval.
