# Supplementary figures and images for: Preconception syphilis seroprevalence and association with duration of marriage and age among married individuals in Guangdong Province, China: A population-based cross-sectional study
Source: PLoS Negl Trop Dis. 2022 Nov 28;16(11):e0010884. doi: 10.1371/journal.pntd.0010884 (PMC9731487; doi:10.1371/journal.pntd.0010884)

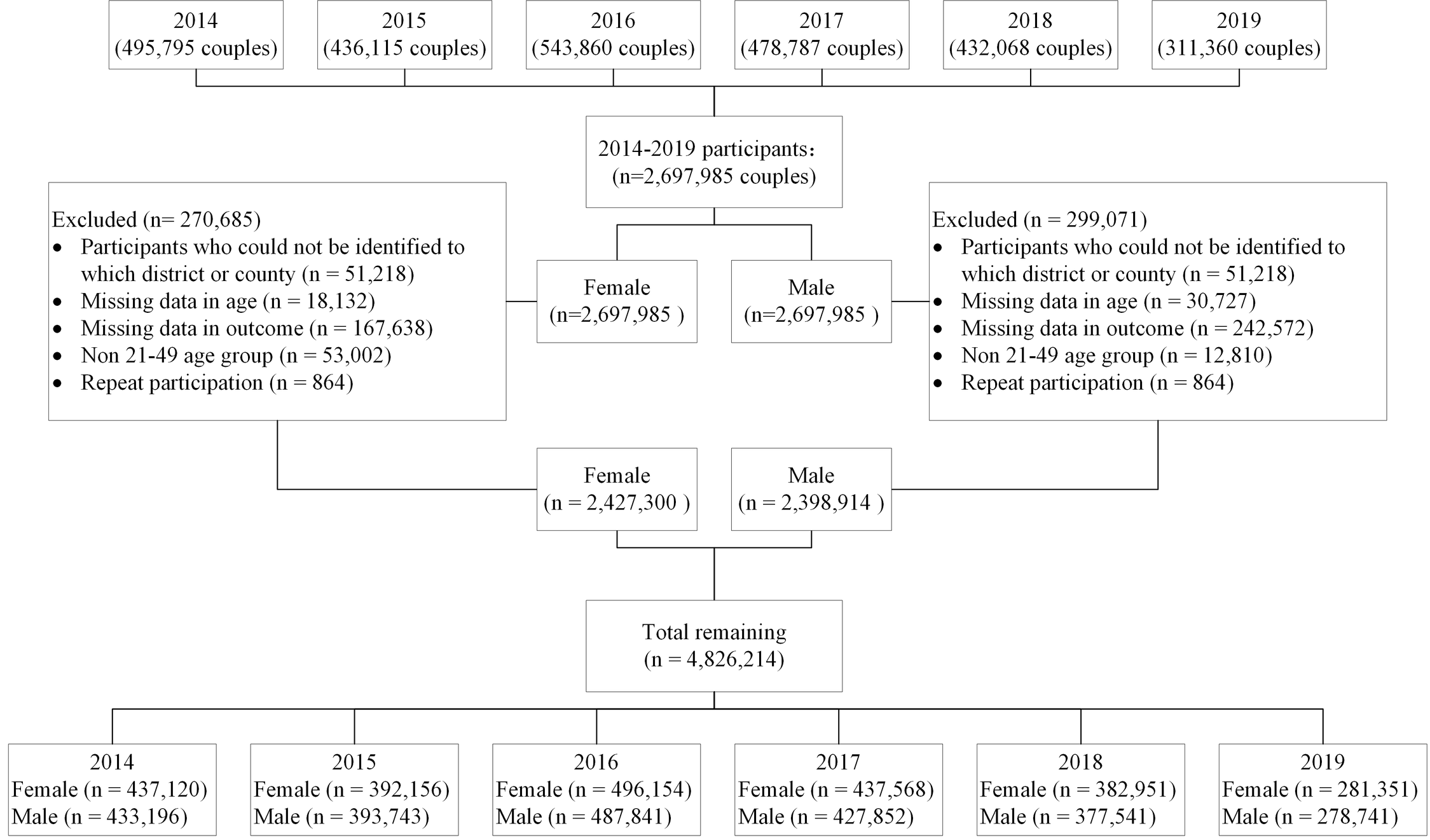

Supplement: S1 Fig — (TIF) [file pntd.0010884.s006.tif]

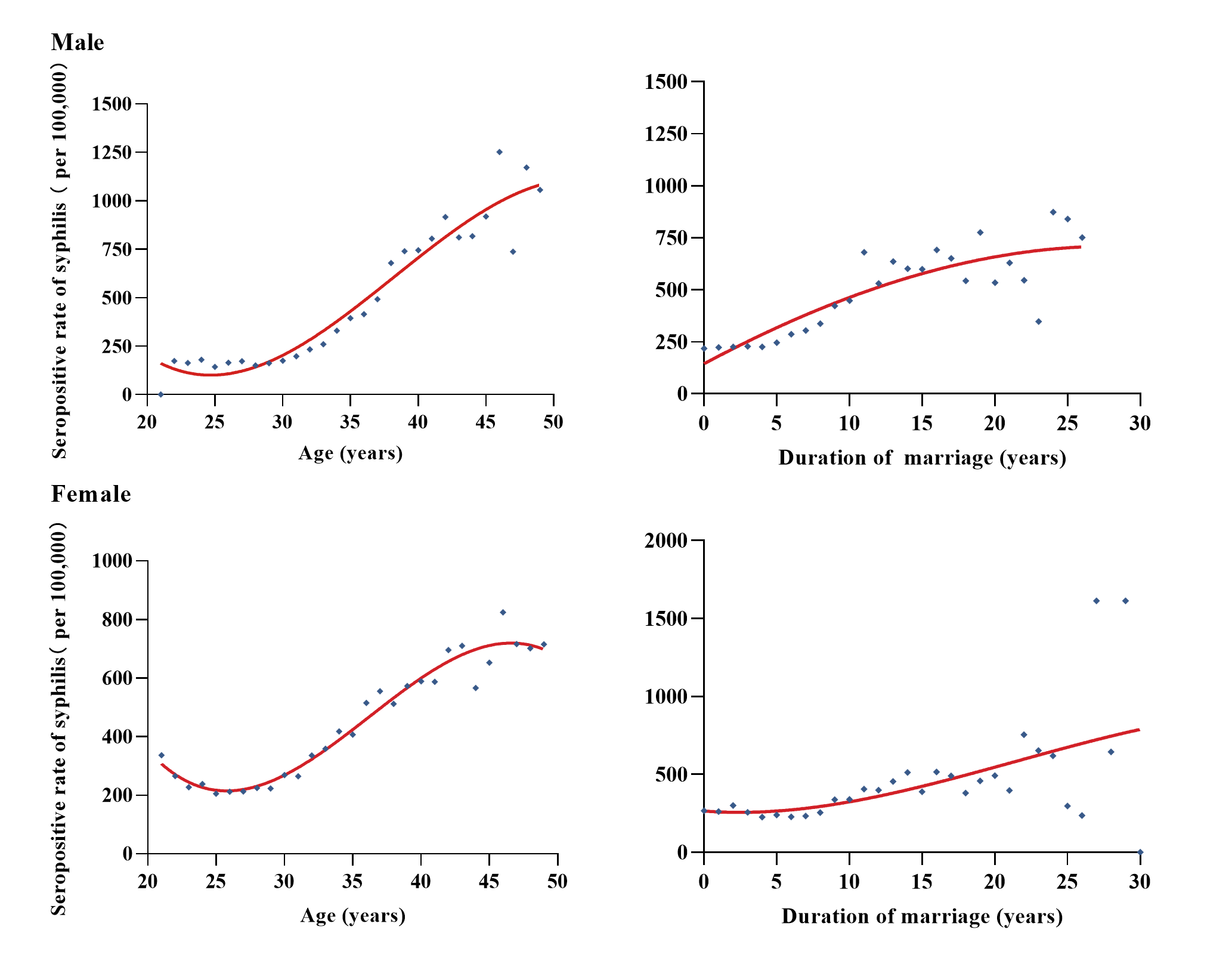

Supplement: S2 Fig — Seroprevalence of syphilis was calculated as the sum of all seropositive cases divided by the sum of participants over the period 2014–2019, multiplied by 100,000. The curve is fitted by a third-order polynomial. (TIF) [file pntd.0010884.s007.tif]

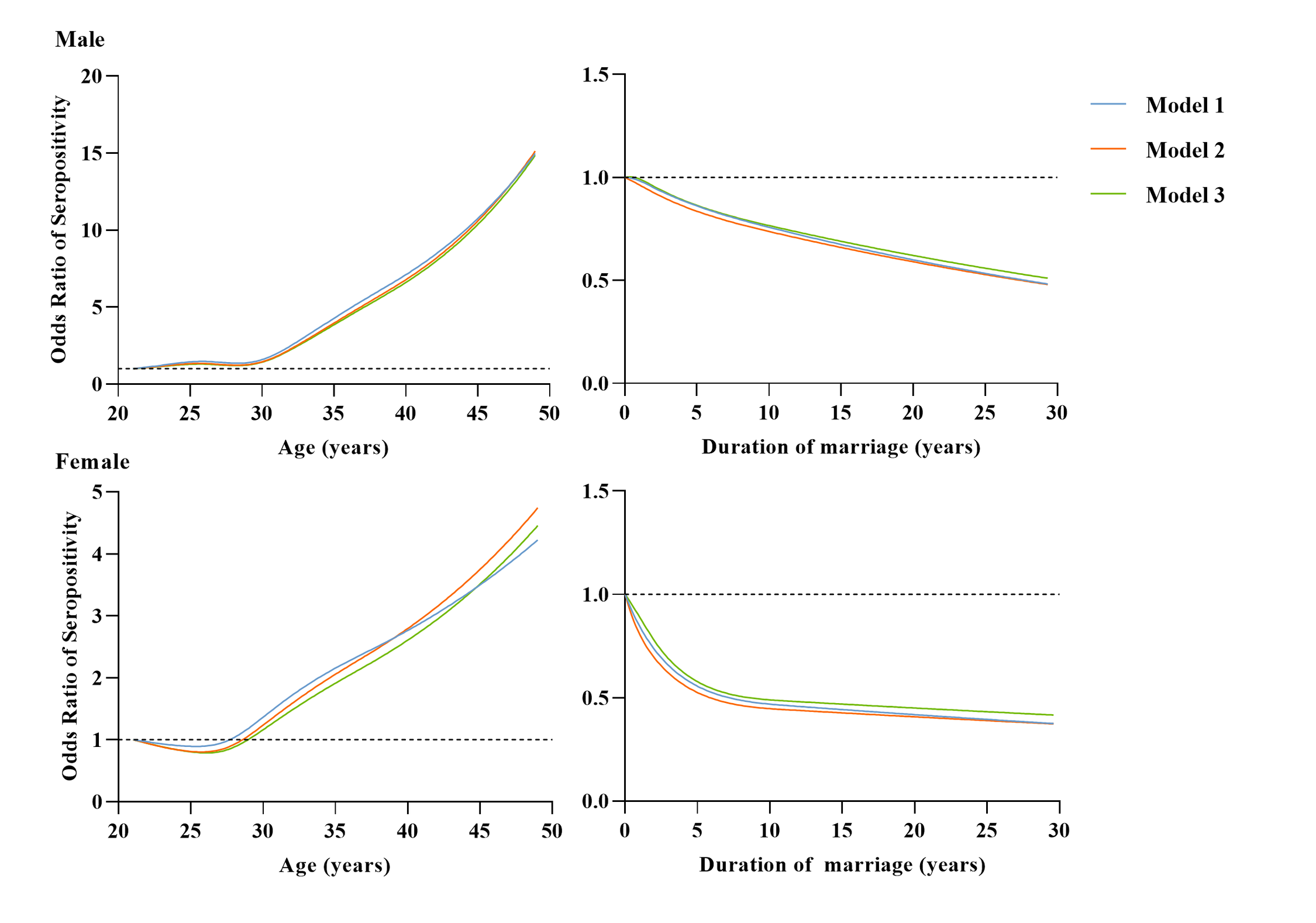

Supplement: S3 Fig — Adjusted for ethnicity, education, occupation, household registration, migrant, smoking, drinking, ever used drug, syphilis seropositivity of spouses and region and pregnancy history (only for women). Duration of marriage and age were mutually adjusted in the restricted cubic splines models with five knots and the reference points were the respective minimum values. Model 1: no adjustment for occupation; Model 2: no adjustment for educational level; Model 3: no adjustment for occupation and educational level. (TIF) [file pntd.0010884.s008.tif]

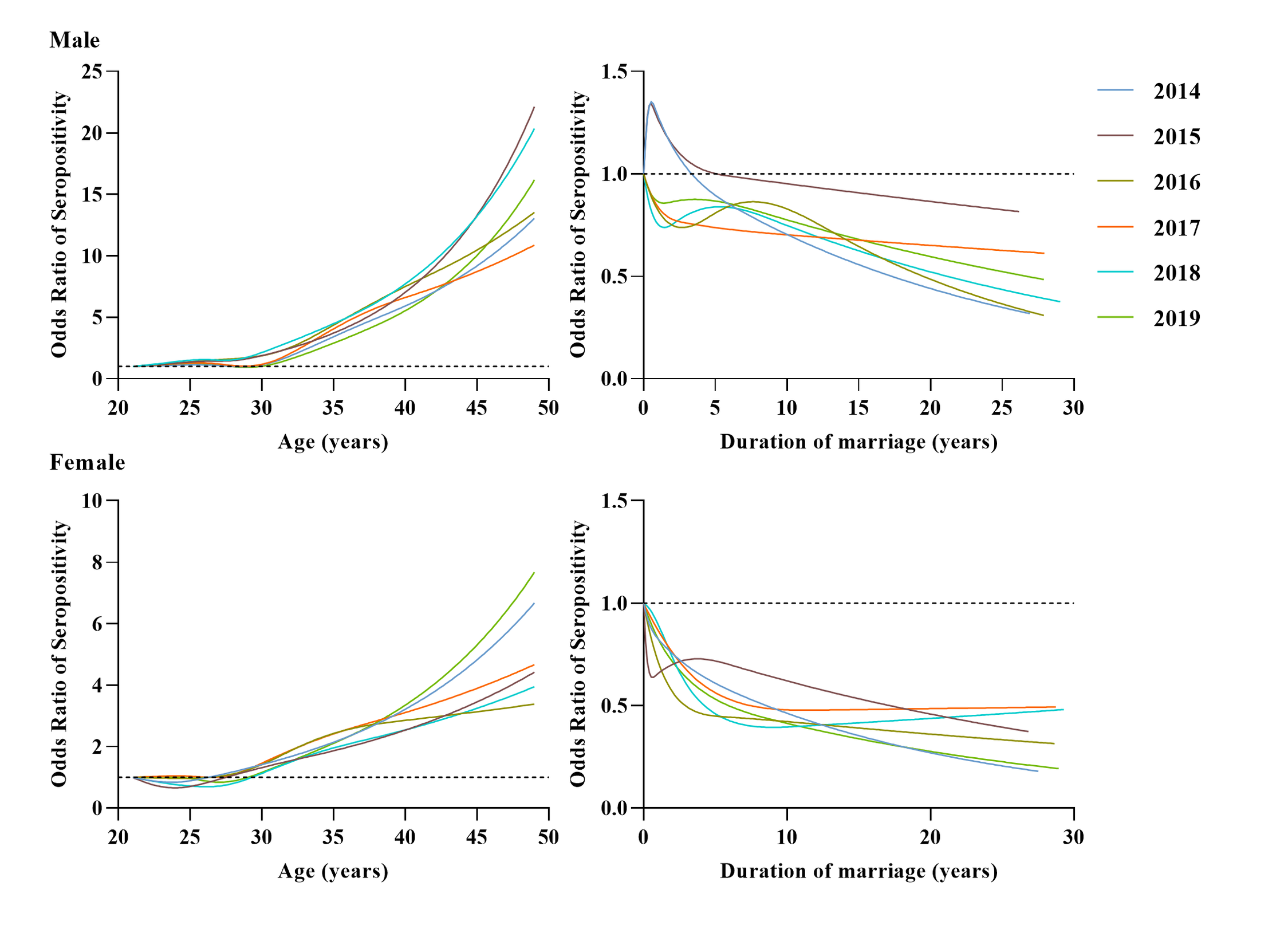

Supplement: S4 Fig — All models were adjusted for ethnicity, education, occupation, household registration, migrant, smoking, drinking, ever used drug, syphilis seropositivity of spouses and region and pregnancy history (only for women). Duration of marriage and age were mutually adjusted in the restricted cubic splines models with five knots and the reference points were the respective minimum values. (TIF) [file pntd.0010884.s009.tif]

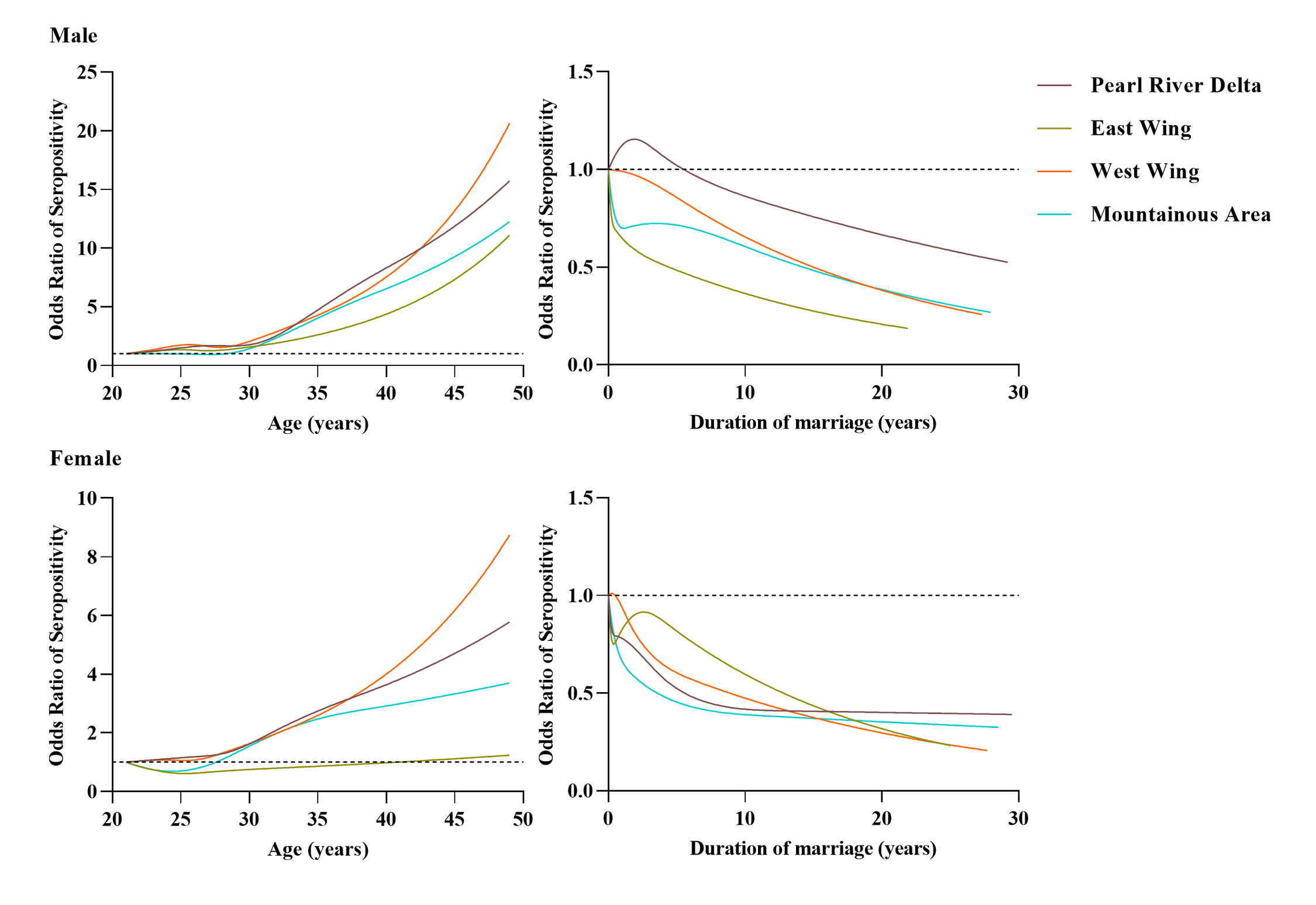

Supplement: S5 Fig — All models were adjusted for ethnicity, education, occupation, household registration, migrant, smoking, drinking, ever used drug, syphilis seropositivity of spouses and pregnancy history (only for women). Duration of marriage and age were mutually adjusted in the restricted cubic splines models with five knots and the reference points were the respective minimum values. (TIF) [file pntd.0010884.s010.tif]

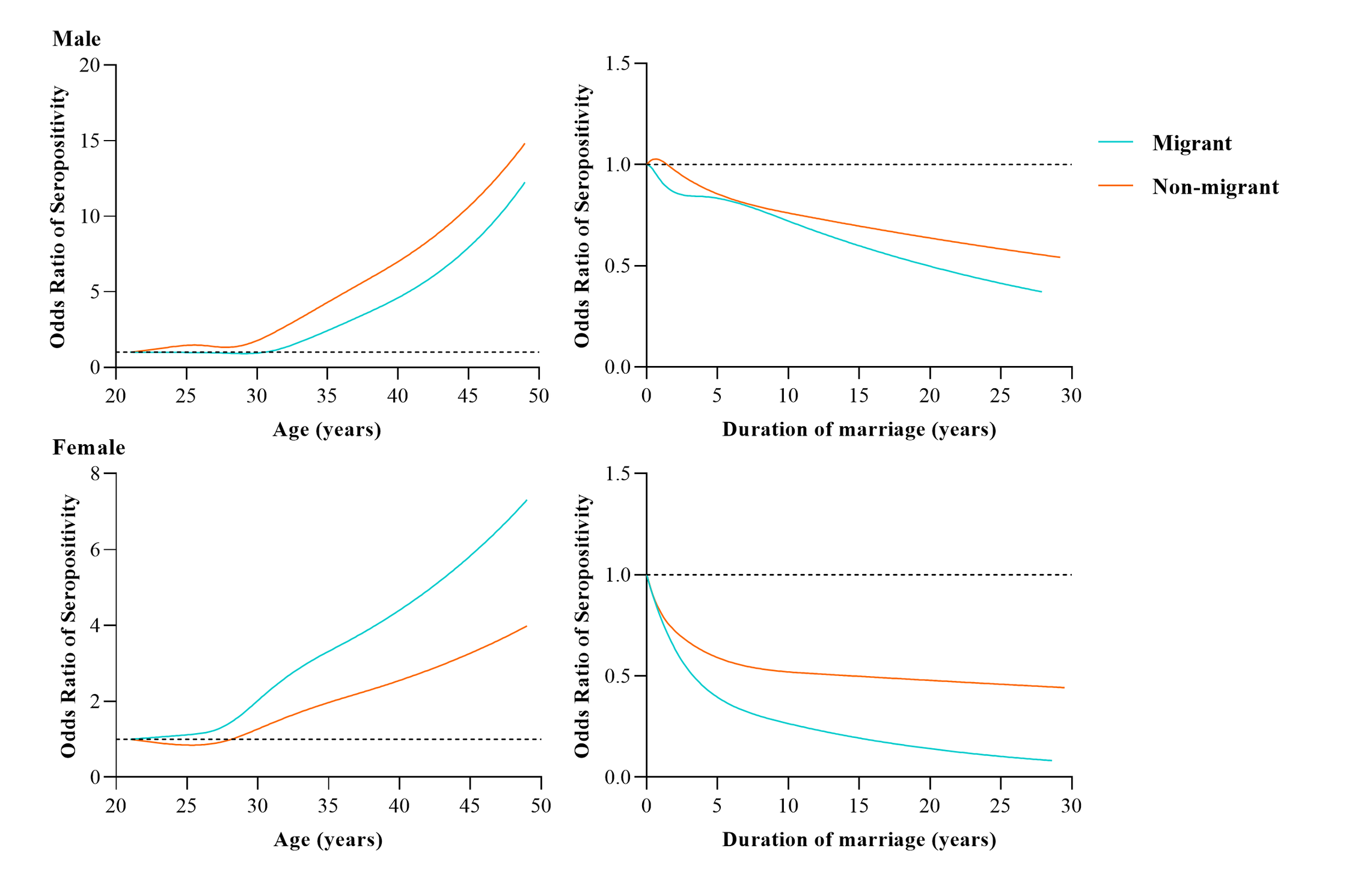

Supplement: S6 Fig — All models were adjusted for ethnicity, education, occupation, household registration, smoking, drinking, ever used drug, syphilis seropositivity of spouses and region and pregnancy history (only for women). Duration of marriage and age were mutually adjusted in the restricted cubic splines models with five knots and the reference points were the respective minimum values. (TIF) [file pntd.0010884.s011.tif]
