# Supplementary material for: Preconception syphilis seroprevalence and association with duration of marriage and age among married individuals in Guangdong Province, China: A population-based cross-sectional study
Source: PLoS Negl Trop Dis. 2022 Nov 28;16(11):e0010884. doi: 10.1371/journal.pntd.0010884 (PMC9731487; doi:10.1371/journal.pntd.0010884)
Supplement: Attachment — Submitted filename: Response to reviewers 0810.docx [file pntd.0010884.s012.docx]

Dear editor,

Thank you very much for your letter and all the comments and suggestions on our manuscript entitled “*Syphilis seroprevalence and association with duration of marriage and age among married individuals aged 21-49 years in Guangdong Province, China: A population-based cross-sectional study*” (PNTD-D-22-00684). We also thank the reviewers for the constructive comments and suggestions. We do appreciate the opportunity to revise and resubmit the manuscript. Several modifications were made in response to the reviewers’ comments and hopefully the new version could be qualified to be published. Thank you again for the valuable comments!

Yours sincerely,

Li Ling

**Editor’s comments:**

**Q1:** In addition, please consider more clearly articulating the clinical importance of your findings given that the key strategy for preventing MTCT of syphilis is antenatal screening of women during pregnancy.

Response1: Thank you for the constructive comments.

Screening for syphilis in asymptomatic, nonpregnant persons who were at increased risk for infection is an important approach to preventing the sexual transmission of syphilis and subsequent mother-to-child transmission (MTCT) (1). When deciding who to screen for syphilis, clinicians should know the prevalence of syphilis infection in the communities they serve and other sociodemographic factors that may be associated with an increased risk of syphilis infection (1).

In this study, we found the highest syphilis seroprevalence (SSP) in less developed areas within Guangdong Province. This suggests that in resource-limited areas, guaranteeing the support in terms of financial subsidy, clinical professional training, disease surveillance, and screening of high-risk groups might make sustainable progress in the prevention of MTCT of syphilis (2). Second, individuals who are newly married and in their late 20s or older are at increased risk of syphilis. These results emphasize the necessity to screen for syphilis in newly married individuals who are in their late 20s or older. Moreover, in clinical practice, medical professionals could advise pregnant women’s male spouses to undergo syphilis testing as well.

We have added the related information in *Author summary* (Line 63-65 and Line 75-79), *Introduction* (Line 91-96), *Discussion* (Line 485-496) and *Conclusion* (538-545) section.

References:

1.	US Preventive Services Task Force USPSTF, Bibbins-Domingo K, Grossman DC, Curry SJ, Davidson KW, Epling JW, Jr., et al. Screening for Syphilis Infection in Nonpregnant Adults and Adolescents: US Preventive Services Task Force Recommendation Statement. JAMA. 2016;315(21):2321-7.

2.	Tang X, Chen W, Tang SQ, Zhao PZ, Ling L, Wang C. The evaluation of preventive and control measures on congenital syphilis in Guangdong Province, China: a time series modeling study. Infection. 2022.

**Reviewers’ comments:**

**Reviewer #1:** Thank you for the invitation to review this manuscript.

This study has a good idea to describe the seroprevalence and spatiotemporal distribution of syphilis and to investigate the association of duration of marriage (DoM) and age with syphilis seroprevalence (SSP).

However, there are several minor comments need to be addressed.

**Q1:** Page 9 Line 134: Information on duration of marriage was collected, it would be better to display the distribution of this variable in the manuscript.

**Response1**: Thank you for the comments. We agree with the reviewer’s suggestion and have displayed the distribution of the duration of marriage (Line 278-279 and Table 1).

**Q2**: Page 12 Line 201: To examine the independent association of DoM and age with SSP, why not adjust such potential confounding variables like ethnicity, educational, occupation?

**Response2:** Sorry for the unclear presentation. In these models, we first adjusted potential confounding variables, including ethnicity, education, occupation, household registration, migrant, smoking, drinking, ever used drug, syphilis seropositivity of spouses, and region (Line 226-228). Then, to examine the independent association of DoM with SSP, we adjusted for these potential confounding variables and age. To examine the independent association of age with SSP, we adjusted for these potential confounding variables and DoM.

We have revised this sentence to “*To examine the independent association of DoM and age with SSP, we* *additionally adjusted for DoM or age in the above multivariate models*” (Line 231).

**Q3:** Page 17 Table 1: The distribution of variables among different age group is showed. Maybe it would be better to demonstrate the total distribution of variables in both sex. Moreover, the distribution of DoM among different age group in specific sex is varied, which could cause potential selection bias to your conclusion made in the same specific sex.

**Response3:** Thank you for the constructive comments.

We agree with the reviewer’s suggestion and have changed the Table 1 to demonstrate the total distribution of variables in both sex (Line 289). The distribution of variables among different age group was also showed in *Supplementary Table 2* and *Supplementary Table 3*.

We agree with the reviewer’s comment. The National Free Preconception Health Examination Project (NFPHEP) provided free preconception screening for syphilis to legally married couples planning a pregnancy within the next six months. However, married individuals who did not plan to have a baby during the study period were excluded, which may lead to the selection bias the reviewer mentioned, which is a limitation of this study. As we stated in the Method section, this project was well implemented in Guangdong Province, with a coverage rate of over 85% of the target population (1) (Line 140). Preconception health care is also recommended in the United States, while the preconception health care rate is about 50% (2).

We have added the related information in the *Strengths and limitations* section (Line 525-531).

References:

1.	Guangdong Medical Information Institute, Guangdong Medical Academic Exchange Center. Guangdong Health and Family Planning Yearbook 2015-18 and Guangdong Health Statistical Yearbook 2019-20 [cited 2022 February 15]. Available from: http://www.gdyqs.cn/channels/559.html.

2.	Curry SJ, Krist AH, Owens DK, Barry MJ, Caughey AB, Davidson KW, et al. Screening for Syphilis Infection in Pregnant Women: US Preventive Services Task Force Reaffirmation Recommendation Statement. JAMA. 2018;320(9):911-7.

**Q4:** Page 17 Table 1: The proportion of participants with a 0 year duration of marriage is relatedly large. Does this mean this group of people are all newly married couples and the duration of marriage is less than one month? It is better to be explained in notes.

**Response4:** Sorry for the confusion. In this study, duration of marriage referred to the time difference (in years) between syphilis screening and marriage registration (Line 201). In the data processing, we retained one decimal place. Participants with a 0 year duration of marriage are newly married couples, and the duration of marriage is no more than 18 days (18/365 ≈ 0.049 ≈ 0 year). Moreover, the duration of marriage between 19 and 365 days was considered to be “0.1-1.0 years”.

We have explained these concepts in notes as suggested (Line 291-294 and Line 344-347).

**Q5:** Page 30 Line 473: Have you showed the results of subgroup analyses for four regions?

**Response5:** Thank you for the comments. We have presented the results of subgroup analyses for four regions in *Supplementary* *Figure 5* (Line 722).

**Reviewer #2:** This is a population-based epidemiological study focused on reproductive married individuals to find the association of DoM(Duration of marriage) and age with SSP (syphilis seroprevalence). The study is interesting. However, the conclusions remain to be discussed.

**Q1:** The authors used RPR test as screening SSP. How can they avoid the serological false positive or false negative results of RPR?

**Response1:** Thank you for the constructive comments.

Quality assurance is critical to avoid serological false positive or false negative results, especially for the RPR test, which was subjected to many factors (1). In the National Free Preconception Health Examination Project (NFPHEP), syphilis serology testing was performed by trained and qualified health workers in local laboratories affiliated with medical institutions under qualified quality control mechanisms. Test kits approved by the National Medical Products Administration were selected by local laboratories according to their preferences. As a national project, quality control was performed regularly with the following control measures: a) establishing the commission of the National Center of Clinical Laboratories for Quality Inspection and Detection, including experts from chemical, clinical, microbiological, and immunological laboratories; b) convening of committees to discuss critical factors that may affect laboratory results and draft quality control plans for key items; c) conducting external quality assessments twice a year, covering 13 items (including the results of the RPR test). At each randomly selected site, five samples were randomly selected. The scoring system was as follows: five correct was 100 points, four correct was 80 points, and less than four correct was unqualified (1, 2). Scores ≥80 were scaled as excellent. The excellent rate was reported to be over 96% (3). Moreover, health workers would inform the seropositive couples and refer them to a specialist in sexually transmitted diseases for further confirmation.

We have added the related information in *Method* (Line 164-179), and *Strengths and limitations* (Line 513-521) section.

References:

1.	Zhou Q, Yang Y, Wang L, Chen X, Xu Q, Wang Q, et al. Intra-couple discordance in preconception syphilis screening for both spouses: a national and population-based survey in China, 2013-2018. BJOG. 2022;129(2):313-21.

2.	Zhang SK, Wang QM, Shen HP. Design of the National Free Preconception Health Examination Project in China (in Chinese). Natl Med J Chin. 2015;95(3):162-5.

3.	Zhou Q, Zhang S, Wang Q, Shen H, Tian W, Chen J, et al. China’s community-based strategy of universal preconception care in rural areas at a population level using a novel risk classification system for stratifying couples´ preconception health status. BMC Health Services Research. 2016;16(1).

**Q2:** The main conclusion of this study is that the higher the positive rate of syphilis serum with age. This conclusion may be biased by RPR screening. RPR can be positive for life even after syphilis treatment. Therefore, the newly infected syphilis and the treated syphilis cause the positive rate of RPR to increase with age.

**Response2:** Thank you for the constructive comments.

We agree with the reviewer’s comment that the newly infected syphilis and the treated syphilis cause the positive association between syphilis seroprevalence (SSP) and age.

Both the newly infected syphilis and the treated syphilis in pregnant women should be identified and managed (1), because even after treatment, the risk of adverse pregnancy outcomes remains significantly higher compared to uninfected pregnancies (1). In addition, the reinfection rate of syphilis is high (2), with 10.94% reported in Shenzhen (3), 9.17% reported in Belgium (4), and 7.3% reported in Florida (5). All stages of syphilis in pregnant women poses a risk of MTCT and the risk is highest in primary and secondary stages, followed by the early latent stage (6). Transmission risk continuous during the first 4 year after exposure, after which the risk of vertical transmission declines over time (6). It was recommended that pregnant women with previous syphilis infection should be screened again at 28 weeks of gestation and at delivery (7). Therefore, even though the RPR test cannot distinguish the treated or the newly infected infection, our findings still have important clinical and public health implications.

We found that the association between age and SSP was not statistically significant in younger individuals (in early 20s), and age was positively associated with SSP in older individuals (Figure 4, Line372). These results emphasize the necessity to screen for syphilis in newly married individuals who are in their late 20s or older. Additionally, early syphilis prevention strategies should be implemented among young people as early as possible to mitigate the increasing cost of syphilis to the health system.

We have added the related information in *Discussion* section (Line 427-438).

References:

1.	Rac MW, Revell PA, Eppes CS. Syphilis during pregnancy: a preventable threat to maternal-fetal health. Am J Obstet Gynecol. 2017;216(4):352-63.

2.	Hsu KK, Molotnikov LE, Roosevelt KA, Elder HR, Klevens RM, DeMaria A, Jr., et al. Characteristics of cases with repeated sexually transmitted infections, Massachusetts, 2014-2016. Clin Infect Dis. 2018;67(1):99-104.

3.	Luo Z, Zhu L, Ding Y, Yuan J, Li W, Wu Q, et al. Factors associated with syphilis treatment failure and reinfection: a longitudinal cohort study in Shenzhen, China. BMC Infect Dis. 2017;17(1):620.

4.	Kenyon C, Osbak KK, Crucitti T, Kestens L. Syphilis reinfection is associated with an attenuated immune profile in the same individual: a prospective observational cohort study. BMC Infect Dis. 2018;18(1):479.

5.	Matthias J, Sanon R, Bowen VB, Spencer EC, Peterman TA. Syphilitic Reinfections During the Same Pregnancy-Florida, 2018. Sex Transm Dis. 2021;48(5):e52-e5.

6.	Peeling RW, Mabey D, Kamb ML, Chen XS, Radolf JD, Benzaken AS. Syphilis. Nat Rev Dis Primers. 2017;3:17073.

7.	Ghanem KG, Ram S, Rice PA. The Modern Epidemic of Syphilis. N Engl J Med. 2020;382(9):845-54.

**Q3:** In Table 2, the author divided the marriage age into three groups: 0, 0-1.0, >1.0 (years). What does the first group of 0 mean?

**Response3:** Sorry for the confusion. In this study, duration of marriage referred to the time difference (in years) between syphilis screening and marriage registration (Line 201). In the data processing, we retained one decimal place. Participants with 0 year duration of marriage (the first subgroup in Table 2) were newly married couples and the duration of marriage was no more than 18 days (18/365 ≈ 0.049 year ≈ 0 year). We have revised the second subgroup to “0.1-1.0”, and the duration of marriage between 19 and 365 days was considered to be “0.1-1.0 years”.

We have added the related information in notes (Line 291-294 and Line 344-347).

**Q4:** The authors should explain why in male group, they used two statistical models and got the different results on the relationship between marriage age and RPR positive rate.

**Response4:** Thank you for the constructive comments.

As shown in Table 2 (Line 341), we used two statistical models to examine the relationship between duration of marriage (DoM) and syphilis seroprevalence (SSP) and got the opposite results in male group. This may be due to the fact that age was an important confounding factor in the association of DoM and SSP in males. As we stated in the *Results* section, we calculated the Spearman correlation between DoM and age, which was 0.49 in males and 0.53 in females (Line 316). This implies that age and DoM is positively correlated. Moreover, age was strongly associated with SSP (Line 323). Therefore, in model 1, without adjustment for age, it showed that DoM was a risk factor. The results for model 1 and 2 were similar in females, which may be due to the slightly weaker association between age and SSP in females compared to males (Table 2).

We have added the related information in the *Discussion* section. (Line 449-456).
