# Supplementary material for: Preconception syphilis seroprevalence and association with duration of marriage and age among married individuals in Guangdong Province, China: A population-based cross-sectional study
Source: PLoS Negl Trop Dis. 2022 Nov 28;16(11):e0010884. doi: 10.1371/journal.pntd.0010884 (PMC9731487; doi:10.1371/journal.pntd.0010884)
Supplement: Attachment — Submitted filename: Response letter.docx [file pntd.0010884.s013.docx]

Manuscript number: PNTD-D-22-00684R1

Manuscript title: *Preconception syphilis seroprevalence and association with duration of marriage and age among married individuals in Guangdong Province, China: A population-based cross-sectional study*

Date: 10 October 2022

Dear Editor Michael Marks,

Thank you very much for your letter regarding our manuscript entitled “*Preconception syphilis seroprevalence and association with duration of marriage and age among married individuals in Guangdong Province, China: A population-based cross-sectional study*” (Manuscript number: PNTD-D-22-00684R1).

We appreciate the opportunity to revise and resubmit our manuscript. We have revised the manuscript based on reviewers’ suggestions and comments under track mode (please see the details below).

**Reviewers’ comments:**

**Methods:**

**Reviewer #2**: (No Response)

**Reviewer #3**: I am unable to assess the robustness of geospatial techniques and analyses mentioned in the methods but I am able to assess the suitability and interpretation of regression models.

**Point1**: Line 154: Are these community workers also considered health workers in the hospital system? Or would these include individuals within the communities/districts? Furthermore, how are couples who are planning on a pregnancy identified (e.g., outreach to districts, through community organizations?)

**Response**: We apologize for the unclear expression. The community workers mentioned in our manuscript are actually health workers who work at the family planning service center (1, 2). (Line 166)

Couples who are planning a pregnancy are volunteering to participate in the National Free Preconception Health Examination Project (NFPHEP). In order to recruit targeted participants, the government set up counseling stations at marriage registration offices, held lectures in the communities/districts, and used various forms of media, such as television and newspapers, to reach these couples. The NFPHEP has also been implemented by providing special staff to inform couples of the project when they apply for the Family Planning Service Certificate, which is a must if couples plan to have children in China (3). Then those couples who come forward to consult on the project and follow fertility policy are enrolled by health workers at the family planning services center (2).

We have added the above content in the *Method* section (Line 159-167).

Reference:

1.	Wu SC. Family planning technical services in China. Front Med China. 2010;4(3):285-9.

2.	Zhang SK, Wang QM, Shen HP. Design of the National Free Preconception Health Examination Project in China (in Chinese). Natl Med J Chin. 2015;95(3):162-5.

3.	People’s Government of Guangdong Province. Guangdong Province, Family Planning Service Certificate Management Methods. [cited 2022 October 1]. Available from: http://www.gd.gov.cn/zwgk/wjk/zcfgk/content/post_2532346.html.

**Point2**: It remains slightly unclear if both spouses were approached to get tested as well as asked to complete separate questionnaires - could this be clarified in the study design?

**Response**: Thank you for pointing this out. The NFPHEP provided free syphilis screening test for both spouses and sociodemographic characteristics of each spouse were obtained by different questionnaires.

We have clarified this in the *Method* section as suggested (Line 170 and 174).

**Point3:** Line 197: For "syphilis seropositivity of spouses" - could the authors clarify if this was a question asking participants about whether they knew if their spouse had syphilis? Furthermore, is this a history of Syphilis or ongoing infection? More details on what this means would help to clarify the inclusion of this covariate.

**Response**: Thank you for the constructive comments. The variable “syphilis seropositivity of spouses” refers to the test results of rapid plasma reagin of their spouses, and a positive result means either a historical or an ongoing syphilis infection (1). Since a population-based study in rural China showed that the syphilis seropositivity of spouses was strongly associated with syphilis seropositivity (2), we included this variable in models.

We have added the related information in the *Method* section (Line 211-213).

Reference:

1.	Ghanem KG, Ram S, Rice PA. The Modern Epidemic of Syphilis. N Engl J Med. 2020;382(9):845-54.

2.	Liao KJ, Zhang SK, Liu M, Wang QM, Liu J, Shen HP, et al. Seroepidemiology of Syphilis Infection among 2 million reproductive-age women in rural china: A Population‑based, Cross‑sectional Study. Chinese Medical Journal. 2017;130(18):2198-204.

**Results**:

**Reviewer #2**: (No Response)

**Reviewer #3**: The analysis presented matches the analysis plan and are clearly presented.

**Conclusions**:

**Reviewer #2**: (No Response)

**Reviewer #3**: The conclusions are supported by the data present. I have several questions on limitations:

**Point1**: Would the authors agree that the method of sampling (community workers identifying couples) have any selection biases?

**Response**: Thank you for your comments and we agree with your opinion. This limitation has been added to the *Strengths and limitations* section (Line 537).

**Point2**: Would including both spouses in analysis lead to some bias (assuming maybe couple-level correlations).

**Response**: We thank you for raising this question. Both of the logistic models and the restricted cubic splines in this study were stratified by gender to address couple-level correlations (1) (Line 234-235). Since there is no requirement for independence in the hot spot analysis (2), we included both spouses in the hot spot analysis. Therefore, the results of this study may not be biased by couple-level correlations.

Reference:

1.	Liao KJ, Zhang SK, Liu M, Wang QM, Liu J, Shen HP, et al. Seroepidemiology of Syphilis Infection among 2 million reproductive-age women in rural china: A Population‑based, Cross‑sectional Study. Chinese Medical Journal. 2017;130(18):2198-204.

2.	Chang BA, Pearson WS, Owusu-Edusei K, Jr. Correlates of county-level nonviral sexually transmitted infection hot spots in the US: application of hot spot analysis and spatial logistic regression. Ann Epidemiol. 2017;27(4):231-7.

**Editorial and Data Presentation Modifications**

**Reviewer #2**: (No Response)

**Reviewer #3**: I only had minor comments for this manuscript. Unfortunately, I am unfamiliar with a majority of statistical approaches used in this paper, so I had provided minor comments on the study design and limitations. However, it seems like the previous reviewers have provided robust comments in these aspects.

**Summary and General Comments**

**Reviewer #2**:

**Point1**: Over all what the authors replies were not satisfied me. For example. The answer to Question1 is far from satisfactory, for RPR can be positive or negative in many situations. RPR alone can’t confirm syphilis. The authors did not answer my concerns in Question 2.

**Response**: We apologize for not answering your concerns in the previous revision. We thank you very much for your reconsideration and have provided detailed answer to the Questions 1 and 2 (please see the details below).

***Question 1: The authors used RPR test as screening SSP. How can they avoid the serological false positive or false negative results of RPR?***

**Response**: We agree that RPR alone can’t confirm syphilis. All the positive samples would be further confirmed by the treponemal test (i.e., *Treponema pallidum* particle agglutination test) (1). However, in the NFPHEP, information on confirmatory tests was not collected, which was a limitation of this study (Line 523). Nevertheless, the sensitivity of the RPR test is 86%, 100%, and 98% for primary, secondary, and latent syphilis, respectively, and the specificity is about 98% (2). There were other studies that used RPR test results for analysis: a study used PRP test results to investigate syphilis seropositivity and intra-couple discordance among married couples planning a pregnancy in China (3). Further well-designed studies, using the same brands of diagnostic kits and the same laboratory teams, are needed to delimit cases of false negatives and positives.

We have added the related information in the *Strengths and limitations* section (Line 523-533).

Reference:

1.	Zhang SK, Wang QM, Shen HP. Design of the National Free Preconception Health Examination Project in China (in Chinese). Natl Med J Chin. 2015;95(3):162-5.

2.	Cantor AG, Pappas M, Daeges M, Nelson HD. Screening for Syphilis: Updated Evidence Report and Systematic Review for the US Preventive Services Task Force. JAMA. 2016;315(21):2328-37.

3.	Zhou Q, Yang Y, Wang L, Chen X, Xu Q, Wang Q, et al. Intra-couple discordance in preconception syphilis screening for both spouses: a national and population-based survey in China, 2013-2018. BJOG. 2022;129(2):313-21.

***Question 2: The main conclusion of this study is that the higher the positive rate of syphilis serum with age. This conclusion may be biased by RPR screening. RPR can be positive for life even after syphilis treatment. Therefore, the newly infected syphilis and the treated syphilis cause the positive rate of RPR to increase with age.***

**Response**: We apologize for the unclear expression. What we would like to conclude is that syphilis screening among married individuals aged ≥25 years old should be recommended (Line 59), not that older people are at higher risk of new syphilis infection. Because both the newly infected syphilis and the treated syphilis should be identified and well-managed (1). Previous studies showed that the reinfection rate of syphilis is high (2), and even after treatment, the risk of adverse pregnancy outcomes remains significantly higher compared to uninfected pregnancies (1).

We have added the related information in the *Discussion* section (Line 443-453).

Reference:

1.	Rac MW, Revell PA, Eppes CS. Syphilis during pregnancy: a preventable threat to maternal-fetal health. Am J Obstet Gynecol. 2017;216(4):352-63.

2.	Matthias J, Sanon R, Bowen VB, Spencer EC, Peterman TA. Syphilitic Reinfections During the Same Pregnancy-Florida, 2018. Sex Transm Dis. 2021;48(5):e52-e5.

**Point2**: The influencing factors of fetal syphilis mainly depend on the maternal prenatal screening and proper and prompt treatment of syphilis, not RPR alone. I think the manuscript is of little significance to prevent congenital syphilis and to update the knowledge of clinicians.

**Response**: We thank you for your comments. The maternal prenatal screening remains inadequate for eliminating congenital syphilis (1). A national study showed that in China, the preconception syphilis seroprevalence among women was 0.37% (2), but the incidence of syphilis was 0.025% (3). The pre-exiting syphilis infection accounts for a large proportion of syphilis infection. To further eliminate maternal fetal transmission and reduce the risk of adverse pregnancy outcomes, preconception syphilis screening is considered an option and is recommended by both the US Preventive Services Task Force and the US Center for Disease Control and Prevention (CDC) (4, 5). When deciding who to screen for syphilis, clinicians should know the prevalence of syphilis in the communities they serve and other sociodemographic factors that may be associated with an increased risk of syphilis infection (4). Therefore, we explored the seroprevalence and spatiotemporal distribution of syphilis among married individuals planning to conceive in Guangdong Province, China and investigated the high-risk groups. The results of this study revealed high-risk areas in Guangdong Province and provided the latest baseline data for the development of a better prevention and control program for syphilis. This study also supported recommendations for comprehensive screening of syphilis before pregnancy.

We have added the above content in the *Introduction* (Line 91-101) and *Discussion* section (Line 494-496).

Reference:

1.	Zhou Q, Yang Y, Wang L, Chen X, Xu Q, Wang Q, et al. Intra-couple discordance in preconception syphilis screening for both spouses: a national and population-based survey in China, 2013-2018. BJOG. 2022;129(2):313-21.

2.	Liao KJ, Zhang SK, Liu M, Wang QM, Liu J, Shen HP, et al. Seroepidemiology of Syphilis Infection among 2 million reproductive-age women in rural china: A Population‑based, Cross‑sectional Study. Chinese Medical Journal. 2017;130(18):2198-204.

3.	Wang AL, Qiao YP, Wang LH, Fang LW, Wang F, Jin X, et al. Integrated prevention of mother-to-child transmission for human immunodeficiency virus, syphilis and hepatitis B virus in China. Bull World Health Organ. 2015;93(1):52-6.

4.	US Preventive Services Task Force USPSTF, Bibbins-Domingo K, Grossman DC, Curry SJ, Davidson KW, Epling JW, Jr., et al. Screening for Syphilis Infection in Nonpregnant Adults and Adolescents: US Preventive Services Task Force Recommendation Statement. JAMA. 2016;315(21):2321-7.

5.	Gao R, Liu B, Yang W, Wu Y, Wang B, Santillan MK, et al. Association of Maternal Sexually Transmitted Infections With Risk of Preterm Birth in the United States. JAMA Netw Open. 2021;4(11):e2133413.

**Reviewer #3**: (No Response)

**Figure Files:**

While revising your submission, please upload your figure files to the Preflight Analysis and Conversion Engine (PACE) digital diagnostic tool, https://pacev2.apexcovantage.com. PACE helps ensure that figures meet PLOS requirements. To use PACE, you must first register as a user. Then, login and navigate to the UPLOAD tab, where you will find detailed instructions on how to use the tool. If you encounter any issues or have any questions when using PACE, please email us at [figures@plos.org](mailto:figures@plos.org).

**Response**: Done as required.

**Data Requirements:**

Please note that, as a condition of publication, PLOS' data policy requires that you make available all data used to draw the conclusions outlined in your manuscript. Data must be deposited in an appropriate repository, included within the body of the manuscript, or uploaded as supporting information. This includes all numerical values that were used to generate graphs, histograms etc. For an example see here: <http://www.plosbiology.org/article/info%3Adoi%2F10.1371%2Fjournal.pbio.1001908#s5>.

**Response:** The data analyzed in our study are not publicly available because of the limitation of data availability in the data management rule of Guangdong Provincial Fertility Hospital. Access to these data may be requested through the Guangdong Provincial Fertility Hospital, China (contact via sysuhanlu@126.com) for researchers who meet the criteria for access to confidential data.

**Reproducibility:**

To enhance the reproducibility of your results, we recommend that you deposit your laboratory protocols in protocols.io, where a protocol can be assigned its own identifier (DOI) such that it can be cited independently in the future. Additionally, PLOS ONE offers an option to publish peer-reviewed clinical study protocols. Read more information on sharing protocols at <https://plos.org/protocols?utm_medium=editorial-email&utm_source=authorletters&utm_campaign=protocols>

**Response:** Not applicable.

Thank you again for the time and effort you and the reviewers dedicated to providing these insightful comments. We wish that the revised version could be qualified for publication.

Yours sincerely,

Li ling
